# Supplementary material for: Maternal Uniparental Disomy of Chromosome 20 (UPD(20)mat) as Differential Diagnosis of Silver Russell Syndrome: Identification of Three New Cases
Source: Genes (Basel). 2021 Apr 17;12(4):588. doi: 10.3390/genes12040588 (PMC8073552; doi:10.3390/genes12040588)
Supplement: Supplementary file 1 [file genes-12-00588-s001.zip › genes-1164878-supplementary.pdf]

# Supplemental Information for:

## Maternal Uniparental Disomy of Chromosome 20 (UPD(20)mat) as Differential Diagnosis of Silver Russell Syndrome: Identification of Three New Cases

Pierpaola Tannorella [1], Daniele Minervino [1], Sara Guzzetti [1], Alessandro Vimercati [1], Luciano Calzari [1], Giuseppa Patti [2,3], Mohamad Maghnie [2,3], Anna Elsa Maria Allegri [2], Donatella Milani [4], Giulietta Scuvera [4,5], Milena Mariani [6], Piergiorgio Modena 7, Angelo Selicorni [6], Lidia Larizza [1] and Silvia Russo [1,*]

1. Research Laboratory of Medical Cytogenetics and Molecular Genetics, IRCCS Istituto Auxologico Italiano, 20095 Milan, Italy
2. Department of Pediatrics, IRCCS Istituto Giannina Gaslini, 16147 Genova, Italy;
3. Department of Neuroscience, Rehabilitation, Ophthalmology, Genetics, Maternal and Child Health (DINOGMI), University of Genova, 16132 Genova, Italy
4. Pediatric Highly Intensive Care Unit, Fondazione IRCCS Ca' Granda Ospedale Maggiore Policlinico, 20122 Milan, Italy;
5. Medical Genetics Unit, Woman-Child-Newborn Department, Fondazione IRCCS Ca' Granda-Ospedale Maggiore Policlinico, via Francesco Sforza 28, 20122 Milan, Italy
6. UOC Pediatria, ASST Lariana, 22100 Como, Italy;
7. SOS-ID Laboratorio di Genetica, ASST Lariana, 22100 Como, Italy; piergiorgio.modena@asst-lariana.it

**Correspondence:** Silvia Russo, Research Laboratory of Medical Cytogenetics and Molecular Genetics, IRCCS Istituto Auxologico Italiano, 20095 Milan, Italy. Tel.: +39-0261-9113-036, Email: s.russo@auxologico.it

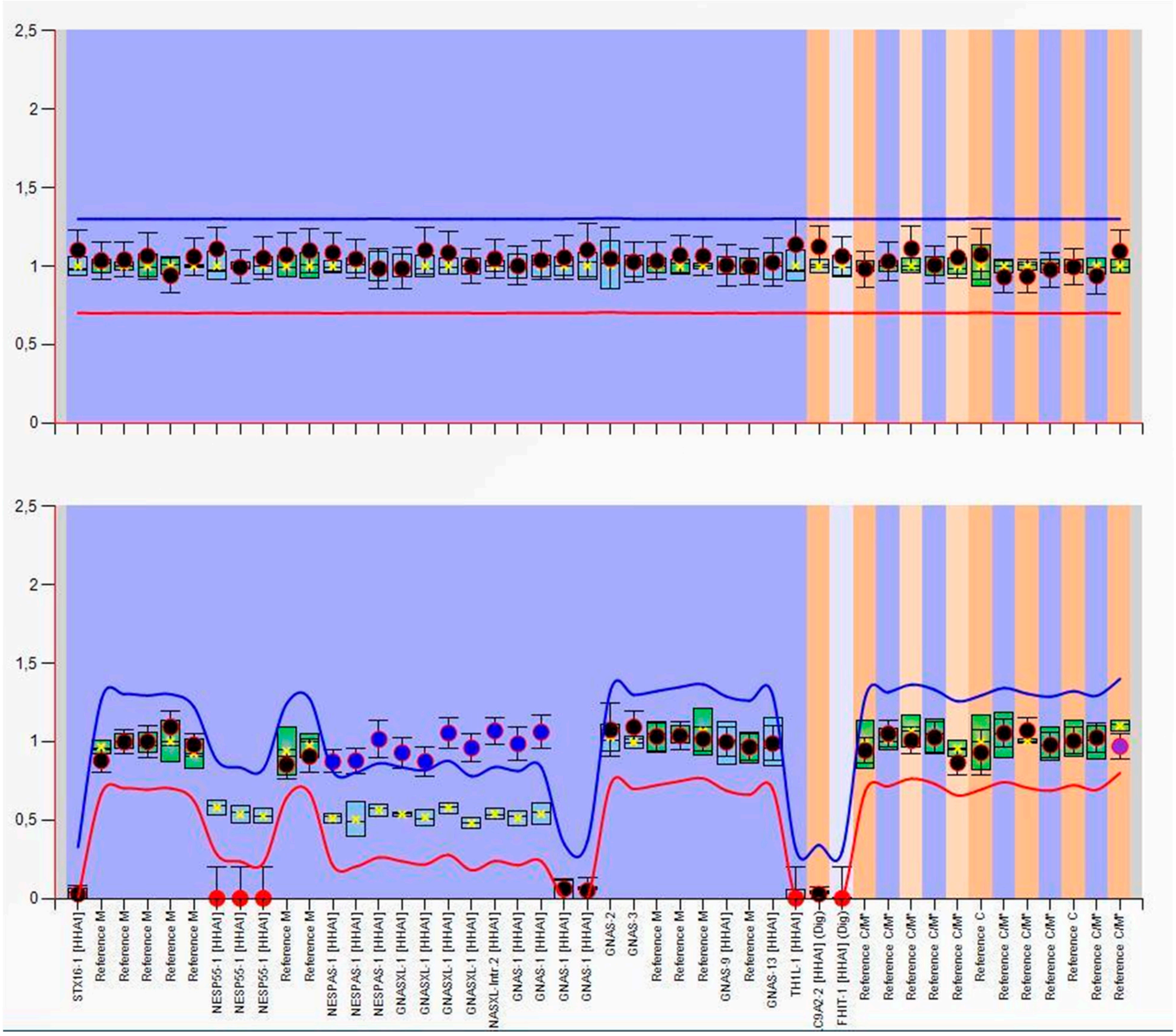


**Figure S1**: MS-MLPA plot of a patient with UPD(20)mat. Analyses of the *GNAS* MS-MLPA (ME031) shows normal results for the copy numbers (top panel) and aberrant methylation at *GNAS* locus(bottom panel) in all patients; specifically, a complete hypomethylation of *GNAS-NESP:TSS-DMR* and complete hypermethylation of the other three DMR on *GNAS* locus *(GNAS-AS1*:TSS-DMR, *GNAS-XL*:Ex1-DMR, and *GNAS A/B*:TSS-DMR).
